# Rare primary bladder mucosa-associated lymphoid tissue lymphoma: A case report and review of literature

**DOI:** 10.3389/fonc.2022.1042459

**Published:** 2023-01-05

**Authors:** Xi Tu, Xiyao Zhuang, Fen Li, Chaoyou Huang, Youliang Qian

**Affiliations:** ^1^ Department of Urology, Chengdu Second People’s Hospital, Chengdu, Sichuan, China; ^2^ Department of Internal Medicine, Chengdu Shuangliu Hospital of Traditional Chinese Medicine, Chengdu, Sichuan, China; ^3^ Department of Pathology, Chengdu Second People’s Hospital, Chengdu, Sichuan, China

**Keywords:** bladder, MALT lymphoma, NHL, diagnosis, treatment

## Abstract

Primary bladder mucosa-associated lymphoid tissue (MALT) lymphoma is an extremely rare bladder tumor. Only scarce reports have been reported. We hereby report a case of an 81-year-old female patient with bladder tumor presenting with frequent urination and dysuria, whose pelvic magnetic resonance imaging (MRI) considered bladder cancer. She underwent transurethral resection of the bladder tumor (TURBT), and histopathology confirmed the mass to be bladder MALT lymphoma. The patient refused further treatment, and no disease recurrence one year after surgery. The current data are insufficient to draw conclusions about the long-term efficacy of treatment for this tumor, regular follow-up is necessary. To further understand the clinical features, pathology, treatment and prognosis of this tumor, we have searched the literature from 1990 to the present, analyzing a total of 64 cases of primary MALT lymphoma.

## Introduction

Primary extranodal non-Hodgkin’s lymphomas (NHLs) comprise approximately 10-20% of all NHL cases (1). The most common site of involvement is the gastrointestinal tract, followed by the head and neck, central nervous system, breast, thyroid, skin, bones, and testes. Primary lymphoma involving the bladder is very uncommon, accounting for less than 1% of all bladder tumors and only 0.2% of extranodal lymphomas (2). Mucosa-associated lymphoid tissue lymphoma is a unique subtype of B-cell non-Hodgkin’s lymphoma, which accounts for 7% to 8% of all B-cell lymphomas (3). Therefore, this tumor can easily be misdiagnosed until histologically confirmed. It needs to be clinically differentiated from other diseases including inflammatory lesions, bladder cancer and infection. There is no consensus on the optimal treatment strategy for primary bladder MALT lymphoma. Clinicians can choose surgery or/and radiation or/and chemotherapy based on the patient’s specific situation and their own clinical experience. Although the prognosis of primary bladder MALT lymphoma is good, the current data are insufficient to draw conclusions about the long-term efficacy of treatment for this tumor, regular follow-up is necessary. The present study reported a case of a bladder MALT lymphoma and reviewed the relevant literature to further understand the clinical characteristics, pathology, treatment and prognosis of primary MALT lymphoma, and to strengthen the awareness of this rare disease.

## Case presentation

**Figure 1:**
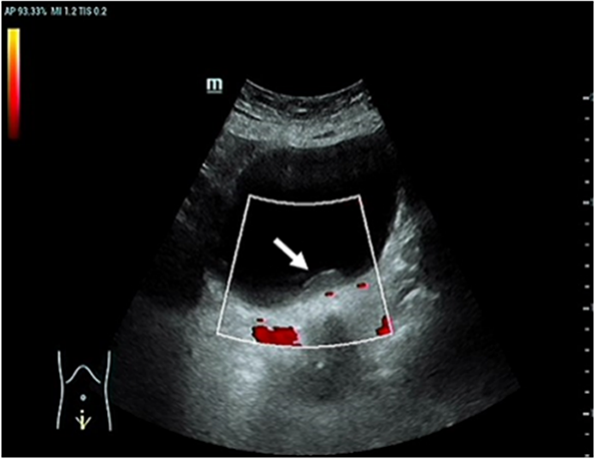
Color ultrasound. This image showed a hypoechoic mass in the posterior wall of the bladder. CDFI: no blood flow signal in the mass [arrow].

**Figure 2:**
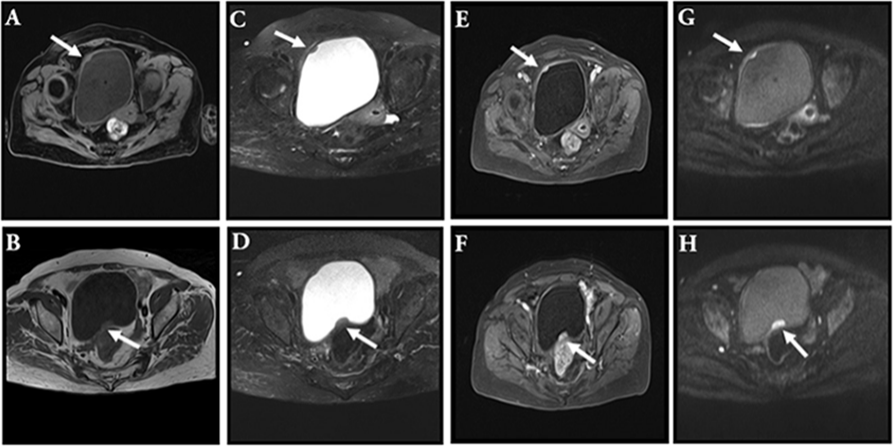
MRI. This image showed a space-occupying lesion with long T1 signal in the right anterior wall of the bladder [**(A)**, arrow]; this image showed a space-occupying lesion with short T2 signal in the right anterior wall of the bladder [**(C)**, arrow]; the enhancement scanning was even [**(E)**, arrow]; diffusion was limited [**(G)**, arrow]. This image showed a space-occupying lesion with long T1 signal in the posterior upper wall of the bladder [**(B)**, arrow]; this image showed a space-occupying lesion with short T2 signal in the posterior upper wall of the bladder [**(D)**, arrow]; the enhancement scanning was even [**(F)**, arrow]; diffusion was limited [**(H)**, arrow].

**Figure 3:**
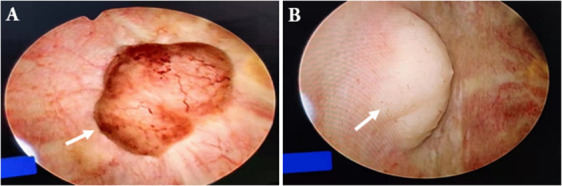
Cystoscope. This image showed a space-occupying lesion in the posterior upper wall of the bladder [**(A)**, arrow]; this image showed a space-occupying lesion in the right anterior wall of the bladder [**(B)**, arrow].

**Figure 4:**
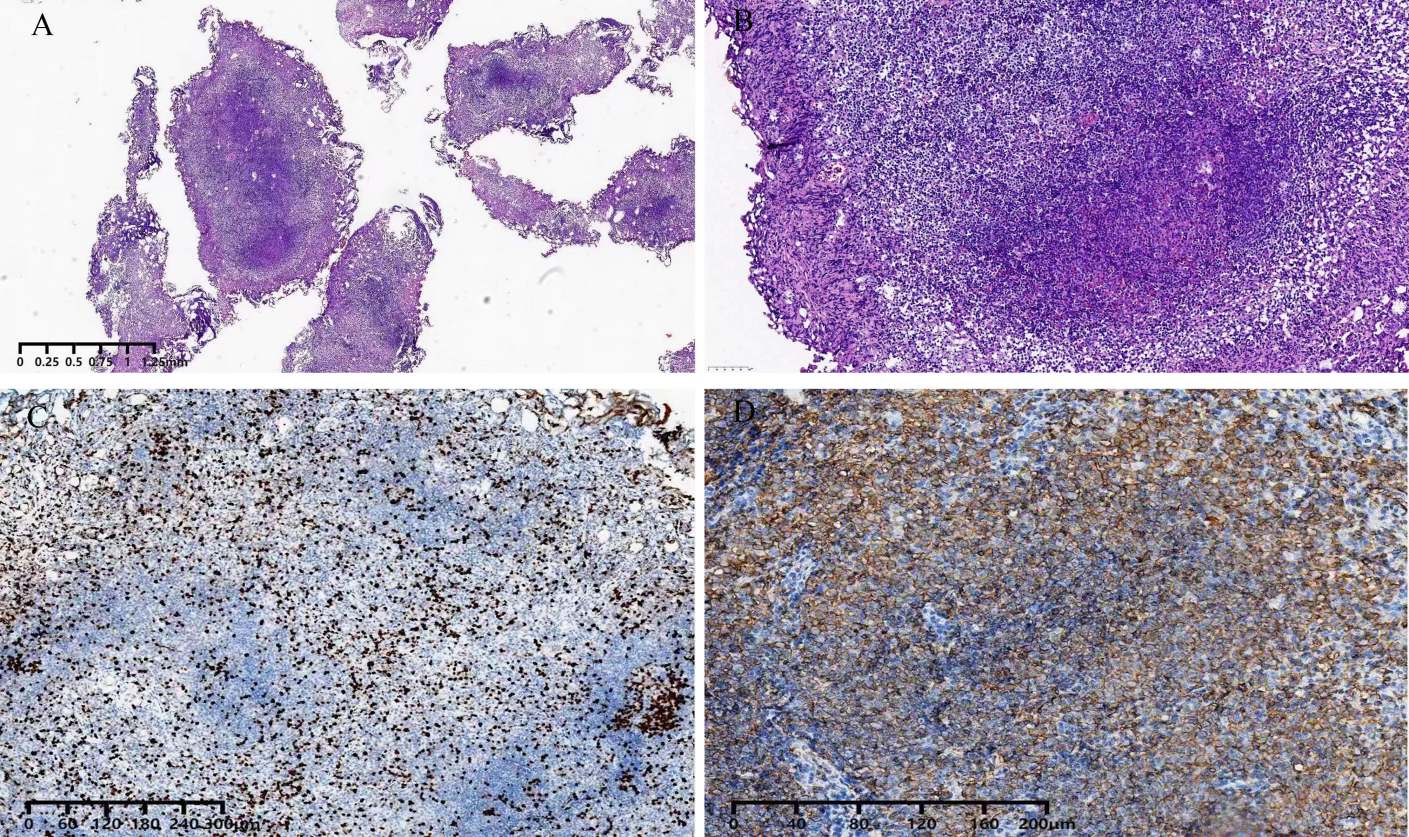
Pathology. Hematoxylin-eosin staining of a surgical specimen. demonstrated MALT lymphoma [**(A, B)**]; Immunohistochemical staining. demonstrated ki-67 expression **(C)**. Immunohistochemical staining demonstrated. CD20 expression **(D)**.

An 81-year-old woman was admitted with symptoms of frequent urination and painful urination. The patient’s symptoms started one month prior to her presentation, and she had no other symptoms. The patient did not undergo relevant examination and treatment. She follows a healthy daily diet, and no relatives in her family had similar illnesses. The patient’s vital signs were normal. Lymph node and abdomen physical examination showed no abnormalities. Urine analysis showed a red blood cell count of 79.4 cells/µl and a white blood cell count of 5071 cells/µl. Urine culture indicated *Escherichia coli*, and other indices were normal. Colour ultrasound showed a 29 mm×9 mm×19 mm hypoechoic mass in the posterior wall of the bladder, with a clear boundary and a regular form. CDFI: no blood flow signal in the mass was noted (**Figure 1**). Pelvic magnetic resonance imaging indicated bladder cancer (**Figures 2A–H**). Computed tomography (CT) of the chest and abdomen revealed no enlarged lymph nodes. The patient underwent transurethral resection of the bladder tumor after the use of antibiotics to control the urinary tract infection. During the operation, the right side and posterior bladder wall showed two nodular lesions with a size of approximately 20 mm×15 mm and 10 mm×10 mm, with a broad base and little bleeding upon resection, respectively (**Figures 3A, B**). Histopathological studies of the resected tumors revealed a large number of proliferating lymphocytes, mainly medium-sized lymphocytes, and some with an empty cytoplasm; lymphoid follicular hyperplasia with irregular enlargement of the marginal area was noted (**Figures 4A, B**). Immunohistochemical studies of the tumors showed that the lesion was positive for the expression of CD20, CD79a, BCL-2, Ki-67 and CD21 and negative for CD3, CD5, CD10, CD23, CD138, cyclin D1 and lambda (**Figures 4C,D**). The pathological diagnosis was consistent with MALT lymphoma. CT scans of the chest and abdomen did not reveal any lymphadenopathy or organ enlargement. Metastatic lesions were not detected. Therefore, the final diagnosis was primary bladder MALT lymphoma. According to the patient’s condition, we recommended the patient to undergo radiotherapy and chemotherapy, but considering the age and general condition of the patient, the patient and family refused further treatment. Therefore, regular follow-up of the patient was recommended. Six months and one year after the operation, no discomfort was reported. No obvious abnormality was detected by cystoscopy. Her follow-up chest and abdominal CT scans showed no abnormality, similar to the previous manifestations. However, the patient was advised to attend lifelong follow-up visits for regular reexaminations.

## Systematic review of literature

The PubMed database was systematically searched for bladder MALT lymphoma from 1990 to 2022. The following keywords were used: (bladder mucosa-associated lymphoid tissue lymphoma) or (bladder MALT lymphoma), and 96 results were retrieved. After excluding secondary bladder mucosa-associated lymphoid tissue lymphoma and review and unrelated studies, 44 articles describing 64 cases (11 men and 53 women) were finally identified. The ratio of male to female was 1:4.8. The mean age at onset was 65 years (range, 17 to 88 years). Twenty-eight of the 37 patients presented with solitary bladder nodules. Twenty-three patients were reported from Asia, of which 18 were from Japan (2, 4–20).28 cases were from Europe, most of them from the United Kingdom (21–27). Including our patient, only four cases had been reported in China (3, 28, 29). All patients had some urinary symptoms at the time of presentation. Most patients presented with hematuria. *Escherichia coli* was the most common pathogen.

There were various treatment strategies for primary bladder MALT lymphoma, including antibiotics, surgery, radiotherapy, chemotherapy and combination therapy. Six patients achieved significant results after antibiotic treatment (11, 13, 25, 30–32). Eleven patients underwent some surgery with or without chemotherapy and/or radiotherapy (3–5, 10, 16, 17, 19, 20, 33–35). The majority of patients (11) received chemotherapy either alone (30) or in combination with radiotherapy (22) (**Table 1**). Hughes et al. reported a patient who was successfully treated with diathermy (26). There was no information on treatment in the three cases. Overall, the treatment effect was good with the longest follow-up time of 156 months.

**Table 1:** Review of characteristics of primary bladder mucosa-associated lymphoid tissue lymphoma.

Cas No.	First author, year	Age(y)/Sex	Symptoms	Tumor number	Urine Culture	Surgery	Follow-up (mo)	Recurrence/ Metastasis	State
1	Hajime K et al.,1990 (4)	56/F	Urinary frequency, micturition pain	Multiple	*Enterococcus*	Total cystectomy	9	No	Japan
2	Pawade J et al.,1993 (21)	67/F	Dysuria	NA	NA	Chemotherapy (CHOP)	24	No	UK
3	Pawade J et al.,1993 (21)	74/F	Dysuria	NA	NA	Radiotherapy	dead	NA	UK
4	Pawade J et al.,1993 (21)	22/F	Hematuria, dysuria, frequency	NA	NA	Chemotherapy and radiotherapy	46	No	UK
5	Pawade J et al.,1993 (21)	83/F	NA	NA	NA	Radiotherapy	20	No	UK
6	Pawade J et al.,1993 (21)	80/M	NA	NA	NA	Not treated	30	Died of unkown cause	UK
7	Fernandez AM et al.,1996 (36)	73/F	Pain left lumbar area, Dysuria	NA	NA	Chemotherapy	8	Died of unrelated cause	Spain
8	Fernandez AM et al.,1996 (36)	50/F	Fever	NA	NA	Chemotherapy	60	No	Spain
9	Fernandez AM et al.,1996 (36)	75/F	Hematuria, Dysuria	NA	NA	Chemotherapy	9	No	Spain
10	Kempton CL et al.,1997 (37)	70/M	NA	NA	NA	Chemotherapy and radiotherapy	48	No	Chile
11	Yuille FA et al.,1998 (22)	80/F	Hematuria	Solitary	NA	Radiotherapy	16	No	UK
12	Gallardo J et al.,1988 (38)	70/F	Hematuria	Solitary	NA	Chemotherapy and radiotherapy	48	NA	Chile
13	Ando K et al.,1999 (5)	77/F	Urinary retention	Solitary	NA	TURBT	36	No	Japan
14	Kawakami K et al.,2000 (6)	27/M	NA	NA	NA	Chemotherapy and radiotherapy	18	No	Japan
15	Tasu JP et al.,2000 (39)	75/F	Hematuria	NA	NA	Chemotherapy and radiotherapy	36	No	France
16	Bates AW et al.,2000 (23)	66/F	NA	Solitary	NA	NA	12	No	UK
17	Bates AW et al.,2000 (23)	79/F	Hematuria	Multiple	NA	NA	NA	NA	UK
18	Bates AW et al.,2000 (23)	59/F	NA	Solitary	NA	NA	36	No	UK
19	Al-Maghrabi J etal.,2001 (40)	64/F	Haematuria, frequency	NA	*Staphylococci, Streptococci, Escherichia coli, Diphtheroid bacillus*	Radiotherapy	156	No	Canada
20	Al-Maghrabi J etal.,2001 (40)	69/F	Frequency, urgency	Multiple	*Escherichia coli*	Radiotherapy	60	No	Canada
21	Al-Maghrabi J etal.,2001 (40)	72/F	Haematuria, nocturia	NA	*Escherichia coli*	Radiotherapy	36	No	Canada
22	Al-Maghrabi J etal.,2001 (40)	62/M	Haematuria, urgency	Solitary	*Staphylococcus aureus*	Radiotherapy	24	No	Canada
23	Wazait HD et al.,2002 (24)	65/F	Dysuria, frequency	NA	*Coliform Bacteria*	Chemotherapy (CHOP)	36	No	UK
24	Wazait HD et al.,2002 (24)	70/F	Hematuria	Solitary	NA	Chemotherapy (Chlorambucil)	60	No	UK
25	Painemal DC et al.,2001 (41)	70/F	Hematuria	Solitary	NA	Chemotherapy and radiotherapy	48	No	Chile
26	vanden-Bosch J et al,2001 (30)	59/M	Hematuria	Solitary	Negative	Antibiotics	36	No	Netherland
27	Krober SM et al.,2002 (31)	57/M	Obstructive dysuria	Solitary	NA	Antibiotics	36	No	Germany
28	Oscier D et al.,2002 (25)	78/F	NA	Solitary	*Escherichia coli*	Antibiotics(trimethoprim, nitrofurantoin, and cephradine)	19	NA	UK
29	Hughes M et al.,2005 (26)	82/F	Hematuria	NA	NA	Chemotherapy (ChlVP)	NA	Died, negative for recurrence	UK
30	Hughes M et al.,2005 (26)	81/F	Hematuria	NA	NA	Diathermy	12	No	UK
31	Hughes M et al.,2005 (26)	28/M	Hematuria	NA	NA	Chemotherapy (ChlVP)	120	No	UK
32	Hughes M et al.,2005 (26)	76/F	Hematuria	NA	NA	Radiotherapy	24	No	UK
33	Hughes M et al.,2005 (26)	77/M	Hematuria	NA	NA	Chemotherapy (ChlD)	48	No	UK
34	Hughes M et al.,2005 (26)	66/F	Recurrent UTIs	NA	NA	Chemotherapy (ChlD)	NA	Died, negative for recurrence	UK
35	Takahara Y et al.,2005 (7)	85/F	Hematuria	Solitary	NA	Radiotherapy	NA	No	Japan
36	Kakuta Y et al.,2006 (8)	84/F	General fatigue, loss of weight	Solitary	NA	Chemotherapy (R-CHOP)	6	No	Japan
37	Hatano K et al.,2007 (9)	84/F	Hematuria	Multiple	*Escherichia coli*	Radiotherapy	14	No	Japan
38	Ueno Y et al.,2007 (10)	64/F	Asymptomatic	Solitary	Negative	TURBT and radiotherapy	19	Recurred in stomach	Japan
39	Fujimura M et al.,2008 (11)	69/F	Hematuria	NA	*Escherichia coli*	Antibiotics	25	No	Japan
40	Terasaki Y et al.,2008 (12)	64/F	General malaise	NA	*Escherichia coli*	retuximab and Radiotherapy	14	No	Japan
41	Sen S et al.,2010 (27)	31/F	Hematuria	Solitary	NA	Chemotherapy (CHOP)	NA	NA	UK
42	Terada T et al.,2011 (13)	88/F	Hematuria	Solitary	NA	Antibiotics	6	NA	Japan
43	Maninderpal KG et al,2011 (4)	65/F	Nausea, feeling unwell	Solitary	NA	Chemotherapy (R-CHOP)	12	dead	Malaysia
44	Szopinski TR et al.,2011 (33)	17/F	Asymptomatic	Solitary	NA	TURBT and chemotherapy	24	No	Poland
45	Morita K et al.,2012 (14)	68/F	Recurrent UTIs	NA	Negative	Chemotherapy (rituximab)	NA	No	Japan
46	Mizuno K et al.,2013 (15)	72/F	Hematuria	Solitary	NA	Chemotherapy (rituximab) and radiotherapy	12	No	Japan
47	Takahashi H et al.,2013 (16)	71/F	Recurrent UTIs	Solitary	NA	TURBT	NA	NA	Japan
48	Bacalja J et al.,2013 (42)	48/F	Asymptomatic	Solitary	*Escherichia coli*	Chemotherapy (R-CHOP), radiotherapy, antibiotics	NA	NA	Croatia
49	Lucioni M et al.,2013 (32)	72/F	Dysuria	Multiple	*Escherichia coli*	Antibiotics (ciprofloxacinfor 6 weeks)	6	No	Italy
50	Chen YR et al.,2014 (28)	63/F	Hematuria	Multiple	Negative	Radiotherapy	11	No	Taiwan
51	Haddad-Lacle JE et al.,2014 (43)	54/M	Asymptomatic	Solitary	Negative	Radiotherapy	36	No	American
52	Matsuda I et al.,2014 (2)	78/F	Cystitis	NA	*Escherichia coli*	Chemotherapy (rituximab)	NA	NA	Japan
53	Hsu JS et al.,2015 (29)	76/F	Recurrent UTIs	Solitary	NA	Radiotherapy	3	No	Taiwan
54	Vempati P et al.,2015 (34)	65/F	Asymptomatic	Multiple	NA	TURBT and radiotherapy	3	No	American
55	Jitani AK et al.,2016 (44)	53/F	Dysuria, urgency, urge incontinence	Solitary	Negative	Chemotherapy (R-CHOP)	6	No	India
56	Ozawa M et al.,2018 (17)	72/F	Asymptomatic	NA	NA	TURBT	13	No	Japan
57	Isono M et al.,2018 (18)	77/F	Pollakisuria, voiding pain, hematuria	Multiple	*Escherichia coli*	Radiotherapy	66	No	Japan
58	Kadam PD et al.,2019 (45)	74/F	Haematuria	Solitary	Negative	Radiotherapy	NA	NA	Singapore
59	Xu H et al.,2020 (3)	77/F	Frequent urination, urinary urgency, dysuria	Multiple	NA	TURBT	15	No	China
60	Lyapichev KA et al.,2020 (46)	58/F	Dysuria, nocturia, urinary frequency	Solitary	NA	Chemotherapy (rituximab)	120	No	American
61	Bhutani N et al.,2020 (35)	40/M	Hematuria	NA	NA	TURBT and radiotherapy and Chemotherapy (R-CHOP)	24	No	India
62	Mandal S et al.,2021 (47)	78/M	Urinary frequency	NA	NA	Radiotherapy	6	No	American
63	Yamamoto A et al.,2021 (19)	74/F	Residual urine	Solitary	NA	TURBT and radiotherapy	12	No	Japan
64	Ishibashi N et al.,2021 (20)	77/F	Hematuria, perineal pain	Solitary	*Escherichia coli*	TURBT and radiotherapy	24	No	Japan

F, female; M, male; y, years old; N/A, not available or provided not in English; UTIS: Urinary tract infection; TURBT, transurethral resection of the bladder tumour; CHOP, cyclophosphamide, doxorubicin, vincristine and prednisolone; R-CHOP, rituximab with CHOP; ChlVP, Chl, chlorambucil; V, vincristine; P, prednisolone; ChlD, Chl, chlorambucil; D, dexamethasone.

## Discussion

Lymphoma is a malignant tumor originating from lymphoid tissue. Due to the absence of lymphoid tissue in various organs of the urinary system, primary lymphoma involving the bladder is extremely rare, which primarily affected older women in our reviews, with a high proportion in Japan and the United Kingdom.

The precise mechanisms of primary bladder MALT lymphoma have not been clarified. Reportedly, 40% of patients with primary bladder lymphoma have a history of chronic cystitis, and *Escherichia coli* is the most common infectious agent (25, 48), which is consistent with the results of our systematic review. In addition, Morita et al. found that autoimmune interstitial cystitis is associated with the development of bladder MALT lymphoma (14).

The common pathological types of bladder lymphoma include low-grade mucosa-associated lymphoid tissue and high-grade diffuse large B cell type of lymphoma (DLBCL), while anaplastic large cell lymphoma (ALCL) is rare. Most primary bladder MALT lymphomas have insidious onset, long course and no typical clinical manifestations. Gross hematuria is the most common clinical symptom, followed by frequent urination, urgency, and dysuria. The differential diagnosis is broad, including inflammatory lesions, bladder cancer, and infection. As a result, patients with this disease are often not diagnosed and treated timely. In addition, some cases of low-grade lymphoma are known to transform into high-grade DLBCL (4), which means that early diagnosis of MALT lymphoma is important to improve prognosis.

Bladder lymphoma usually grows at the base and the trigone of the bladder (35). Commonly, such lesions are initially misdiagnosed as bladder cancer. Imaging examinations usually can not provide much diagnostic information because of their low sensitivity. However, on cystoscopy, these tumors appear as well-defined intracapsular masses typically (33). Diagnosis depends on histopathological and immunohistochemical analyses. Typical MALT lymphoma cells often appear as small to medium-sized lymphocytes with moderate cell size and irregular nuclei, similar to follicular center cells, so they are called “centrocyte-like cells”. Immunohistochemical staining for MALT lymphoma is positive for CD20 and CD79a (3). In this case, the patient’s clinical symptoms, histopathology, and immunophenotype were consistent with MALT lymphoma.

To date, there is no consensus on the optimal treatment strategy for primary bladder MALT lymphoma. There are different therapeutic strategies available for primary bladder MALT lymphoma, depending on the clinical behavior of the tumor, the patient’s general condition, and life expectancy. In all of the cases in our review study, the majority of the patients had presented with localized lesions. A variety of treatment options have achieved a good prognosis. Combined with literature review, it is recommended that TURBT should be attempted first, followed by chemotherapy, or radiotherapy alone, or in combination. In our study, the patient underwent transurethral resection of the bladder tumor to remove the tumor and absence of recurrence at follow-up. All treatment regimens (R-CHOP, CHOP, ChlVP, ChlD) for bladder lymphoma have achieved significant efficacy; R-CHOP was the most frequently used. The R-CHOP chemotherapy regimen has achieved remarkable results in the treatment of both low-grade and high-grade primary bladder lymphomas as monotherapy or in combination therapy (35). Rituximab (anti-CD20 monoclonal antibody) has been shown to be effective in MALT lymphomas with response rates of 55% to 73% (49). Radiotherapy can be used for initial treatment, especially for low-grade or adjuvant treatment after resection (35). Although the prognosis of primary bladder MALT lymphoma is good. It is necessary to regularly follow-up patients who have MALT lymphoma. It must include at least urinary ultrasound and cystoscopy examination. Our reviews indicated that 52 patients were free of recurrence during follow-up of 3 months to 13 years. However, we need to accumulate more cases and long-term follow-up to help better understand the optimal treatment and prognosis of the disease.

## Patient perspective

My frequent and painful urination affected my quality of life and causes me great distress. The doctors helped me make the right diagnosis and chose minimally invasive surgery to completely remove the tumor and eliminated the symptoms of frequent and painful urination. My fear and worry about the tumor disappeared. I achieved physical and psychological healing. I think I’ve been treated very successfully.

## Conclusion

We have described the clinical features, pathology, treatment and prognosis of primary bladder MALT lymphoma to further improve people’s understanding of this rare disease, and primary bladder MALT lymphoma should be included in the differential diagnosis of bladder neoplasm.

## Data availability statement

The original contributions presented in the study are included in the article/supplementary material. Further inquiries can be directed to the corresponding author.

## Ethics statement

The studies involving human participants were reviewed and approved by the Ethics Committee of Chengdu Second People’s Hospital. The patients/participants provided their written informed consent to participate in this study. Written informed consent was obtained from the individual(s) for the publication of any potentially identifiable images or data included in this article.

## Author contributions

XT was the patient’s urologists, reviewed the literature and contributed to manuscript drafting. XZ reviewed the literature and prepared figures. YQ, FL and CH were responsible for the revision of the manuscript for important intellectual content. All authors contributed to the article and approved the submitted version.
